# Supplementary material for: Deep representation learning of electronic health records to unlock patient stratification at scale
Source: NPJ Digit Med. 2020 Jul 17;3:96. doi: 10.1038/s41746-020-0301-z (PMC7367859; doi:10.1038/s41746-020-0301-z)
Supplement: Supplementary file 1 — Supplementary Information [file 41746_2020_301_MOESM1_ESM.pdf]

Supplementary Material

Clustering comparison for the type 2 diabetes analysis

Li et al. [1] used a similar cohort of EHRs as in this study to stratify patients with type 2 diabetes (T2D). Of the $2,472$ patients from their paper, we identified $1,050$ of them in our test sets. To compare the results, we evaluated the similarity of the clusters we obtained to those found by Li et al. via the Fowlkes-Mallows index (FMI), which is an external validation similarity measure of two cluster analyses [2, 3]. FMI scores range from 0 to 1, where 1 represents identical clustering and 0 purely independent label assignments. We obtained FMI $= 0.40$, which suggests that only a portion of patients in groups from Li et al. [1] are identified by our approach as sharing the same characteristics. This may entail that associated clinical phenotypes overlap to a greater extent than hypothesized by Li et al., which may have been overlooked because they collected shorter EHR sequences (i.e., 60 day intervals) and used a manually derived subset of features.

Disease subtyping

Multiple myeloma We identified five subgroups for multiple myeloma (MM) (see Figure 4d and Supplementary Table 7). In particular, subgroup I is characterized by pulmonary manifestations; subgroup II shows bone-related signs of MM; subgroup III includes signs of gastrointestinal problems; subgroup IV is defined by kidney problems; and subgroup V shows signs of peripheral neuropathy.

Pulmunary manifestations in subgroup I include *Pleura effusion*, a rare pulmonary manifestation of amyloidosis [4] that is a comorbidity of MM found in $10-15\%$ of patients (i.e., superimposed amyloidosis). Subgroup I is also characterized by patients with amyloidosis and proteinuria (i.e., excess of proteins in urine) because of the large frequency of *Urea nitrogen* blood test.

Disorders of bone and cartilage largely characterizes patients in subgroup II, which can be identified with bone-related signs of MM.

Subgroups III and V include patients who received chemotherapy and/or anti-cancer medications. In particular, we often found *Bortezomib* in combination with *Dexamethasone* in both subgroups. Bortezomib, for example, is administered to 47% of patients from subgroup III and to 26% of patients in group V. It can be used: 1) for patients ineligible for hematopoietic cell transplantation (HCT); 2) as a maintenance therapy; or 3) in conjunction with HCT for newly-diagnosed patients [5]. Given the characterization of subgroups III and V we expect gastrointestinal problems in subgroup III and *Inflammatory/toxic neuropathy* diagnosis in subgroup V to indicate different side effects from anti-cancer medications. Peripheral nerve damage is also one of the most significant non-hematologic toxicities of Bortezomib [6]. Although unlikely, neurologic complications can also be caused by MM. Such neurologic complications can be due to spinal cord compression from an extramedullary plasmacytoma, or by peripheral neuropathy, which is rare and usually caused by superimposed amyloidosis [7]. The *Counseling* concept in subgroup V likely denotes an encounter to treat severe pain linked to neurologic diseases or psychological support.

Creatinine, *Urea nitrogen*, and *Urinalysis* testing indicate renal function estimate for patients in subgroup IV. Moreover, 9% of patients report *Nephritis and nephropathy* and *Chronic kidney disease* diagnosis, reinforcing the association of subgroup IV to kidney conditions.

Prostate cancer We find 2 subgroups of patients with prostate cancer (PC) related to diverging disease courses (see Figure 4e and Supplementary Table 8).

Clinical manifestation of PC is heterogeneous and may range from asymptomatic screen, microscopic, well differentiated tumor, that may never become clinically relevant; to clinically symptomatic aggressive cancer that causes metastases, morbidity, and death. Treatment approaches for PC include: active surveillance, radical prostatectomy, or radiation therapy (RT) for patients with low-risk PC; prostatectomy or RT in

combination with Androgen Deprivation Therapy (ADT) for patients with higher-risk, but localized PC; RT and ADT for patients with clinical evidence of lymph node involvement.

Patients in subgroup I report *Personal history of PC* and *Ondansetron* medication to prevent RT side effects. This suggests that this group includes patients with recurrent prostate cancer that have either received prostatectomy in the past, and hence RT and ADT is required, or, have already received RT and thus require a radical approach. *Anastomosis* and *Pelvic lymphadenectomy* concepts, which are related to post-prostatectomy procedures and are frequent in these patients, support this description.

Clinical manifestations of PC are usually absent at the time of diagnosis, and over 90% of patients are diagnosed via specific screening (e.g., use of prostate-specific antigen (PSA) or digital rectal examination). Patients in subgroup II show frequent signs of effective PSA screening, indicating probable localized and asymptomatic PC. Diagnosis of *Nocturia*, *Impotence of organic origin*, *Urinary frequency*, and treatments for male sexual dysfunctions, i.e., *Tadalafil, Sildenafil*, are all signs of side effects from PC treatments [8]. Among them, at least 22% likely received a prostatectomy (*Surgery*).

Differently from the second subgroup, patients in the first subgroup do not have PSA among top-ranked concepts. This suggests that subgroup I includes patients that already received prostatectomy, which makes PSA screening less common. Patients in subgroup I appear to have been in the healthcare system for longer and also to have been diagnosed with PC earlier (i.e., similar median age to subgroup II, but absent PSA screening).

Breast cancer Stratification of breast cancer (BC) patients lead to two different subgroups (see Figure 4f and Supplementary Table 9). Subgroup I is linked to advanced stages of BC. Patients in subgroup II, instead, are younger and present a high number of screening-related medical concepts (e.g., *Mammography screening*). In addition, concepts like *Abnormal mammogram* and *Carcinoma in situ of breast* suggest an early-stage diagnosis.

In subgroup I, 23% of patients reports *Unlisted chemotherapy*, with *Surgery* performed on 44% of them. This suggests that these patients may have a more advanced disease, as also evidenced by the lack of screening terms. As a result, they typically undergo chemotherapy treatment, which is more common in advanced stages of BC, whereas primary surgery (lumpectomy, mastectomy), with or without radiation therapy, is preferred for early-stage cancer. This group also includes patients that have already received surgical treatments (33% having received a partial mastectomy) and thus can either be disease free or have relapsed. The presence of *Secondary malignant neoplasm* also suggests that subgroup I includes patients with metastatic BC.

It would be important to better characterize what the general concepts *Unlisted chemotherapy* and *Antineoplastic chemotherapy* specifically refer to in terms of more specific treatments (e.g., hormonal drugs, immunotherapy) to better understand the clinical characteristics of the different subgroups. Moreover, because different molecular subtypes of BC have been identified based on gene expression profiling [9], including hormonal profiles of patients (not available for this study) might improve the stratification results.

Replication of disease subtyping

In the following, we present the patient stratification results obtained with the second split. As highlighted in Supplementary Figure 3, we found slightly different subgroups only for PC and MM (when compared with the results of the first split).

MM encodings detect 4 instead of 5 subgroups. We found two subgroups showing kidney-related problems, one subgroup reporting signs of chemotherapy treatment side effects (i.e., *Inflammatory/toxic neuropathy*) and one subgroup identified by signs of possible superimposed amyloidosis, i.e., *Disease of salivary glands.*

Patients with PC split into three subgroups, where subgroups II and III appears to be a further refinement of subgroup II identified in the first split. In particular, subgroup III includes significantly younger subjects compared to subgroup II. The presence of *Personal history of PC* suggests that subgroups II includes patients

with relapsing PC. This subgroup is of particular importance for the investigation of treatment effectiveness.

The analysis for the other diseases led to very similar results to those obtained with the first split. In particular, for T2D we identified three subgroups: a group with signs of metabolic syndrome and T2D risk factors, a group with microvascular problems, and a third group showing signs of cardiovascular disorders. Patients with PD separates into two subgroups, with motor and non-motor symptoms, respectively, as previously found. AD and BC are again characterized by three and two subgroups, respectively, with the same clinical profiles previously presented.

References

[1] Li, L. *et al.* Identification of type 2 diabetes subgroups through topological analysis of patient similarity. *Science translational medicine* **7,** 311ra174 (2015).

[2] Fowlkes, E. B. & Mallows, C. L. A Method for Comparing Two Hierarchical Clusterings. *Journal of the American Statistical Association* **78,** 553–569 (1983).

[3] Meilă, M. Comparing clusterings – an information based distance. *Journal of Multivariate Analysis* **98,** 873–895 (2007).

[4] Berk, J. L. Pleural effusions in systemic amyloidosis. *Current Opinion in Pulmonary Medicine* **11,** 324–328 (2005).

[5] Attal, M. *et al.* Lenalidomide, Bortezomib, and Dexamethasone with Transplantation for Myeloma. *New England Journal of Medicine* **376,** 1311–1320 (2017).

[6] Argyriou, A. A., Iconomou, G. & Kalofonos, H. P. Bortezomib-induced peripheral neuropathy in multiple myeloma: a comprehensive review of the literature. *Blood* **112,** 1593–1599 (2008).

[7] Dispenzieri, A. & Kyle, R. A. Neurological aspects of multiple myeloma and related disorders. *Best Practice & Research Clinical Haematology* **18,** 673–688 (2005).

[8] Gore, J. L., Kwan, L., Lee, S. P., Reiter, R. E. & Litwin, M. S. Survivorship Beyond Convalescence: 48-Month Quality-of-Life Outcomes After Treatment for Localized Prostate Cancer. *JNCI: Journal of the National Cancer Institute* **101,** 888–892 (2009).

[9] Sørlie, T. *et al.* Repeated observation of breast tumor subtypes in independent gene expression data sets. *Proceedings of the National Academy of Sciences* **100,** 8418–8423 (2003).

	Split 1		Split 2	
	**Train**	**Test**	**Train**	**Test**
Patients	$741,177$	$751,979$	$740,922$	$751,900$
Median sequence length	41	40	41	40
Subsequences	$3,636,014$	$3,656,238$	$3,644,596$	$3,647,426$
Mean N of subseq per patient (sd)	4.91 (12.13)	4.86 (12.06)	4.92 (12.14)	4.85 (12.06)
Vocabulary size	$32,799$	$32,156$	$32,875$	$32,210$

Supplementary Table 1: Train and test set characteristics.

Complex disorder	Test set 1	Test set 2
Type 2 diabetes	$50,253$	$50,327$
Parkinson's disease	$3,124$	$3,150$
Alzheimer's disease	$3,374$	$3,311$
Multiple myeloma	$1,947$	$1,935$
Prostate cancer	$14,401$	$14,508$
Breast cancer	$8,330$	$8,156$
Crohn's disease	$6,668$	$6,741$
ADHD	$6,510$	$6,312$

ADHD = Attention deficit hyperactivity disorder

Supplementary Table 2: Number of subjects in the complex disorder cohorts.

	Test set 1		Test set 2	
	**Numerosity**	**N clusters**	**Numerosity**	**N clusters**
T2D	$48,688$	3	$48,759$	3
PD	$3,052$	2	$3,071$	2
AD	$3,201$	3	$3,150$	3
MM	$1,884$	5	$1,883$	4
PC	$8,522$	2	$8,645$	3
BC	$7,964$	2	$7,838$	2

T2D = Type 2 diabetes; PD = Parkinson's disease; AD = Alzheimer's disease;

MM = Multiple myeloma; PC = Prostate cancer; BC = Breast cancer

Supplementary Table 3: Complex disorder cohorts and number of subclusters identified via patient stratification.

	Type 2 diabetes		
	**Subgroup I** (N=18,325)	**Subgroup II** (N=22,659)	**Subgroup III** (N=7,704)
Female/Male	9,729/8,585*[a]	11,053/11,602*[a]	3,191/4,510*[a]
Age[1]	64.77 (14.32)*[b]	66.52 (15.13)*[b]	69.42 (11.29)*[b]
ICD-9[2]	Hypertension (401.9) - 67% (38%)***	Pain in limb (729.5) - 16% (50%)**	Coronary atherosclerosis (vessel) (414.00) - 67% (40%)***
	Hyperlipidemia (272.4) - 53% (43%)***	Acute kidney failure (584.9) - 15% (84%)***	Coronary artery atherosclerosis (414.01) - 67% (34%)***
	Chest pain (786.50) - 30% (36%)***(I vs III)	Chronic kidney disease (585.9) - 15% (60%)***	Angina pectoris (413.9) - 47% (47%)***
	Obesity (278.00) - 23% (45%)***	Nephritis and nephropathy (583.81) - 13% (60%)***	Percutaneous transluminal coronary angioplasty (V45.82) - 30% (42%)***
	Hypercholesterolemia (272.0) - 20% (34%)***	Peripheral vascular disease (443.9) - 11% (50%)**	Cardiac dysrhythmias (427.89) - 26% (26%)***
Medication	Metformin - 54% (49%)***	Paracetamol - 55% (64%)***	Acetylsalicylic acid - 66% (30%)***
	Calcium - 28% (47%)***	Oxycodone - 42% (66%)***	Clopidrogel - 41% (39%)***
	Vitamin D - 25% (61%)***	Morphine - 29% (80%)***	Bivalirudin - 36% (60%)***
	Cholesterol - 25% (59%)***	Vancomycin - 25% (88%)***	Lisinopril - 24% (19%)*
	Atorvastatin - 20% (43%)***	Furosemide - 24% (72%)***	Amlodipine - 23% (23%)***
Lab test	Glucose - 54% (30%)***	Creatinine - 77% (55%)***	Hematocrit - 71% (18%)***
	Creatinine - 50% (29%)***	Chloride - 77% (56%)***	Mean corpuscular hemoglobin concentration - 70% (18%)***
	Cholesterol - 40% (38%)***	Urea nitrogen - 77% (56%)***	Chloride - 66% (16%)***
	Triglyceride - 40% (38%)***	Albumin - 63% (60%)***	Troponin I cardiac - 46% (26%)***
	Microalbumin panel - 28% (69%)***	Alkaline phosphatase - 63% (60%)***	Cholesterol - 46% (18%)***
CPT-4	Calcium - 39% (28%)***(I vs II)	Potassium - 69% (66%)***	ECG; interpretation, report - 80% (22%)**
	Hemoglobin A1C - 39% (34%)***(I vs II)	Urea nitrogen - 69% (63%)***	Troponin, quantitative - 38% (21%)***
	Glucose (reagent strip) - 37% (31%)***	Urinalysis - 37% (72%)***	C-reactive protein, high sensitivity - 31% (42%)***
	Lipid panel - 24% (27%)***	Hepatic function panel - 24% (70%)***	Echocardiography, transthoracic - 10% (10%)***
	Lipoprotein, direct measurement - 30% (16%)***	Duplex scan of extremity veins - 14% (75%)***	Radiologic examination, chest - 9% (7%)***

[1] Mean (standard deviation); [2] from ICD-9 on in-group and (total) percentages; [a] Multiple pairwise chi-squared test; [b] Multiple pairwise t-test; $* p < 0.05$, $** p < 0.01$, $*** p < 0.001$

ECG = Electrocardiogram

Supplementary Table 4: Most frequent terms for the three subgroups in the type 2 diabetes cohort. We report top five diagnosis (ICD-9), medications, laboratory tests, and CPT-4 procedures. Each clinical term is followed by in-group and total frequencies. Corrected p-values are reported for significant comparisons between groups.

	Parkinson's disease	
	**Subgroup I** (N=1,368)	**Subgroup II** (N=1,684)
Female/Male	596/771[a]	734/950[a]
Age[1]	70.76 (13.52)*[b]	75.17 (14.11)*[b]
ICD-9[2]	Essential tremor (333.1) - 21% (56%)***	Constipation (564.00) - 29% (66%)***
	Anxiety state (300.00) - 20% (45%)	Other malaise and fatigue (780.79) - 25% (72%)***
	Depressive disorder (311) - 14% (40%)*	Coronary atherosclerosis (414.01) - 17% (94%)***
	Abnormality of gait (781.2) - 14% (32%)***	Dysphagia (787.20) - 14% (77%)***
	Dystonia (333.89) - 11% (57%)***	Abdominal pain (789.00) - 14% (90%)***
Medication	Carbidopa/Levodopa combination - 51% (51%)*	Levodopa - 45% (57%)
	Amantadine - 16% (55%)***	Carbidopa - 45% (58%)***
	Pramipexole - 15% (59%)***	Acetylsalicylic acid - 22% (87%)***
	Rasagiline - 14% (60%)***	Docusate sodium - 19% (85%)***
	Selegiline - 12% (57%)***	Vitamin D - 16% (72%)***
Lab test	Mean corpuscular hemoglobin - 3% (4%)***	Glucose - 60% (97%)***
	Leukocytes - 3% (4%)***	Urea nitrogen - 60% (97%)***
	Mean platelet volume - 3% (4%)***	Creatinine - 59% (97%)***
	Width - 3% (4%)***	Potassium - 59% (97%)***
	Erythrocytes - 3% (4%)***	Sodium - 59% (97%)***
CPT-4	Unlisted psychiatric service or procedure - 25% (47%)	ECG; interpretation, report - 51% (95%)***
	MRI (brain, brain stem) - 13% (36%)***	Urea nitrogen - 48% (96%)***
	Surgery - 11% (24%)***	Creatinine - 45% (96%)***
	CT head/brain - 3% (8%)***	Metabolic panel - 35% (98%)***
	Neuropsychological testing - 2% (36%)	Echocardiography, transthoracic - 11% (97%)***

[1] Mean (standard deviation); [2] from ICD-9 on in-group and (total) percentages; [a] Multiple pairwise chi-squared test;

[b] Multiple pairwise t-test; $*\ p < 0.05$, $**\ p < 0.01$, $***\ p < 0.001$;

CT = Computed tomography; ECG = Electrocardiogram; MRI = Magnetic resonance imaging

Supplementary Table 5: Most frequent terms for the two subgroups in the Parkinson's disease cohort.

	Alzheimer's disease		
	**Subgroup I** (N=399)	**Subgroup II** (N=1,170)	**Subgroup III** (N=1,632)
Female/Male	351/48^{***a}	792/378^{***a}	1,249/381^{***a}
Age[1]	54.45 (22.48)^{**b}	84.96 (9.61)^{**b}	72.51 (19.75)^{**b}
ICD-9[2]	Routine gynecological examination (V72.31) - 53% (50%)***	Dementia w/o behavioral disturbance (294.10) - 39% (58%)***	Constipation (564.00) - 33% (61%)***
	Counseling (V65.40) - 34% (20%)**	Altered mental status (780.97) - 27% (68%)***	Anxiety state (300.00) - 24% (64%)*
	Osteoporosis (733.00) - 28% (17%)$^{***(\text{I vs II})}$	Persistent mental disorders (294.8) - 20% (52%)***	Depressive disorder (311) - 21% (64%)
	Family history of osteoporosis (V17.81) - 28% (21%)**	Dysphagia (787.20) - 16% (52%)***	Dementia, unsp., w/o behavioral disturbance (294.20) - 19% (60%)$^{***(\text{III vs II})}$
	Malignant neoplasm of uterus (179) - 22% (45%)***	Intracranial hemorrage (432.9) - 14% (66%)***	Dementia with behavioral disturbance (294.11) - 10% (44%)***
Medication	Calcium - 25% (12%)$^{*(\text{I vs III})}$	Acetylsalicylic acid - 39% (62%)***	Donepezil - 25% (54%)$^{***(\text{III vs I})}$
	Estradiol - 15% (50%)***	Donepezil - 22% (39%)$^{**(\text{II vs I})}$	Memantine - 16% (62%)**
	Iron - 11% (9%)$^{*(\text{I vs III})}$	Levofloxacin - 27% (67%)***	Docusate sodium - 16% (36%)***
	Norethisterone - 9% (64%)***	Vancomycin - 24% (84%)**	Trazodone - 13% (57%)$^{***(\text{III vs I})}$
	Gardasil - 8% (70%)***	Haloperidol - 21% (68%)***	Zolpidem - 9% (63%)$^{***(\text{III vs I})}$
Lab test	Chlamydia/Gonorrhoeae amplified DNA - 10% (37%)***	Mean corpuscular volume - 74% (50%)***	Leukocytes - 53% (50%)***
	Syphilis (rapid plasma reagin) - 6% (6%)***	Creatinine - 74% (51%)***	Glucose - 53% (50%)***
	HIV 1 - 5% (16%)$^{***(\text{I vs II})}$	Erythrocytes - 74% (50%)***	Erythrocytes - 53% (50%)***
	Hepatitis C virus ab - 4% (11%)	Mean corpuscular hemoglobin concentration - 74% (50%)***	Hematocrit - 52% (50%)***
	Hepatitis B surface antigen - 3% (9%)	Glucose - 74% (49%)***	Mean corpuscular hemoglobin concentration - 52% (49%)***
CPT-4	Psychiatric service/procedure - 24% (17%)$^{***(\text{I vs II})}$	ECG - 73% (57%)***	TSH - 44% (62%)**
	Cytopathology, slides, cervical/vaginal - 19% (44%)***	Partial Thromboplastin Time Test - 66% (72%)***	Urea nitrogen - 43% (49%)***
	MRI brain - 5% (7%)$^{**(\text{I vs III})}$	Creatinine - 64% (53%)***	ECG - 39% (42%)***
	CT procedure - 5% (4%)***	Prothrombin time - 56% (71%)***	Psychiatric service/procedure - 22% (62%)$^{***(\text{III vs II})}$
	Brain imaging, PET - 5% (34%)***	Head CT - 45% (61%)***	Head/brain CT - 20% (38%)***

[1] Mean (standard deviation); [2] from ICD-9 on in-group and (total) percentages; [a] Multiple pairwise chi-squared test; [b] Multiple pairwise t-test; $*\ p < 0.05$, $**\ p < 0.01$, $***\ p < 0.001$;

ECG = Electrocardiogram; ab = antibodies; TSH = Thyroid-stimulating hormone; PET = Positron emission tomography; CT = Computed tomography; MRI = Magnetic resonance imaging

Supplementary Table 6: Most frequent terms for the three subgroups in the Alzheimer's disease cohort.

	Multiple myeloma				
	**Subgroup I** (N=469)	**Subgroup II** (N=138)	**Subgroup III** (N=703)	**Subgroup IV** (N=234)	**Subgroup V** (N=340)
Female/Male	236/233^{a}	74/63^{a}	336/367^{a}	129/105^{**a}	144/196^{**a}
Age[1]	69.67 (14.00)^{**b}	64.38 (15.20)^{b}	63.40 (12.57)^{b}	65.39 (17.10)^{b}	63.86 (12.32)^{b}
ICD-9[2]	Edema (782.3) - 53% (31%)*	Disease of salivary glands (527.9) - 28% (9%)***	Diarrhea (787.91) - 55% (58%)***	Hyperlipidemia (272.4) - 25% (19%)$^{***IV\ vs\ II/V}$	Oth inflammatory/toxic neuropathy (357.89) - 34% (22%)**
	Anemia (285.9) - 47% (31%)***	Disorders of bone and cartilage (733.99) - 17% (5%)***	Nausea (787.02) - 48% (56%)*	Dysuria (788.1) - 15% (13%)$^{*(IV\ vs\ II/V)}$	Unsp inflammatory/toxic neuropathy (357.9) - 24% (18%)**
	Shortness of breath (786.05) - 45% (31%)*	Other malaise and fatigue (780.79) - 9% (2%)***	Antineoplastic chemotherapy (V58.11) - 32% (67%)	Malignant neoplasm of colon (153.9) - 15% (38%)*	Counseling (V65.40) - 22% (15%)*
	Pleura effusion (511.9) - 45% (58%)***	Osteoporosis (733.0) - 5% (3%)$^{*(II\ vs\ III)}$	Neutropenia (288.00) - 28% (67%)***	Nephritis and nephropathy (583.81) - 9% (10%)$^{*(IV\ vs\ II/V)}$	Organ/tissue transplant (V42.9) - 14% (11%)$^{***(V\ vs\ II/III/IV)}$
	Fever (780.60) - 43% (32%)***	Fracture (E887) - 5% (2%)$^{***(II\ vs\ I/III)}$	Organ/tissue transplant (V42.9) - 42% (67%)***	Chronic kidney disease (585.9) - 9% (11%)$^{*(IV\ vs\ II)}$	Antineoplastic chemotherapy (V58.11) - 12% (13%)
Medication	Paracetamol - 69% (42%)**	Vitamin D - 7% (2%)**	Calcium - 66% (54%)***	Calcium - 33% (9%)***	Calcium - 42% (17%)***
	Sodium chloride - 65% (41%)**	Oxycodone - 7% (2%)***	Dexamethasone - 54% (55%)**	Vitamin D - 27% (13%)**	Dexamethasone - 28% (14%)**
	Oxycodone - 48% (36%)***	Fentanyl - 7% (2%)***	Ondansetron - 52% (56%)***	Cholecalciferol - 23% (14%)*	Bortezomib - 26% (17%)***
	Fentanyl - 48% (39%)***	Ergocalciferol - 6% (2%)*	Bortezomib - 47% (64%)***	Ergocalciferol - 23% (12%)*	Iron - 25% (17%)***
	Heparin - 48% (46%)***	Acetylsalicylic acid 81 mg - 6% (2%)*	Aciclovir - 45% (67%)***	Atorvastatin - 16% (27%)*	Acetylsalicylic acid 81 mg - 23% (16%)*
Lab test	Erythrocytes - 83% (28%)***	Hemoglobin - 9% (1%)*	Chloride - 93% (47%)*	Protein - 27% (5%)***	Hematocrit - 91% (22%)*
	Glucose - 83% (28%)***	Lymphocytes - 8% (1%)***	Glucose - 93% (46%)***	Glucose - 24% (4%)***	Platelets - 90% (22%)*
	Urea nitrogen - 83% (28%)***	Leukocytes - 8% (1%)**	Potassium - 93% (47%)***	Erythrocytes - 24% (4%)***	Erythrocytes - 90% (22%)***
	Mean corpuscular hemoglobin - 83% (28%)**	Mean platelet volume - 8% (1%)**	Mean corpuscular hemoglobin - 93% (46%)**	Creatinine - 23% (4%)***	Lymphocytes - 90% (23%)***
	Leukocytes - 81% (27%)$^{***(I\ vs\ II/III/IV)}$	Mean corpuscular hemoglobin concentration - 8% (1%)*	Width - 93% (47%)**	Urea nitrogen - 21% (4%)***	Eosinophils - 89% (23%)***
CPT-4	Blood count - 79% (33%)***	Diagnostic/interventional CT - 36% (7%)*	Calcium - 78% (46%)***	Calcium - 42% (8%)***	Gammaglobulin - 63% (24%)**
	Calcium - 73% (28%)***	PET limited area (Head/neck) - 32% (7%)*	Blood count - 75% (51%)**	ECG; interpretation, report - 30% (8%)***	Albumin - 60% (19%)***
	ECG; interpretation, report - 72% (39%)***	PET-CT (skull base to mid-thigh) - 22% (7%)*	Albumin - 73% (49%)***	Urea nitrogen - 28% (6%)***	Calcium, ionized - 59% (17%)***
	Potassium - 71% (34%)*	Tumor imaging PET-CT - 12% (39%)***	Lactate dehydrogenase - 66% (52%)$^{***(III\ vs\ I/II/IV)}$	Cholesterol - 15% (24%)$^{*(IV\ vs\ I/II/V)}$	Lactate dehydrogenase - 58% (22%)*
	PTT - 71% (43%)**	CT thorax (no contrast) - 9% (3%)***	Bone marrow; biopsy - 57% (59%)***	Urinalysis - 12% (9%)$^{***(IV\ vs\ III/V)}$	Beta-2 microglobulin - 55% (26%)***

[1] Mean (standard deviation); [2] from ICD-9 on in-group and (total) percentages; [a] Multiple pairwise chi-squared test; [b] Multiple pairwise t-test; $^{*}p < 0.05$; $^{**}p < 0.01$; $^{***}p < 0.001$;

ECG = Electrocardiogram; CT = Computed tomography; PET = Positron emission tomography; PTT = Partial tromboplastin time

Supplementary Table 7: Most frequent terms for the five subgroups in the multiple myeloma cohort.

	Malignant neoplasm of prostate	
	**Subgroup I** (N=6,916)	**Subgroup II** (N=1,606)
Age[1]	69.64 (12.98)[a]	69.78 (10.56)[a]
ICD-9[2]	Hyperlipidemia (272.4) - 28% (95%)***	Nocturia (788.43) - 29% (33%)**
	Edema (782.3) - 24% (94%)***	Elevated PSA (790.93) - 18% (27%)***
	Personal history of PC (V10.46) - 20% (97%)***	Impotence of organic origin (607.84) - 18% (35%)***
	Hypertrophy (beging) of prostate (600.00) - 14% (85%)***	Urinary frequency (788.41) - 15% (27%)***
	Hematuria (599.70) - 14% (86%)***	Urinary hesitancy (788.64) - 11% (33%)***
Medication	Paracetamol - 44% (98%)***	Midazolam - 17% (12%)***
	Oxycodone - 40% (98%)***	Tadalafil - 14% (35%)***
	Ondansetron - 33% (97%)***	Sildenafil - 12% (33%)***
	Propofol - 31% (94%)***	Tamsulosin - 10% (12%)***
	Morphine - 30% (99%)***	Testosterone - 8% (28%)
Lab test	Glucose - 66% (96%)*	PSA post-prostatectomy - 17% (25%)***
	Leukocytes - 63% (98%)*	PSA free - 10% (27%)***
	Creatinine - 63% (99%)*	Nitrite - 8% (6%)***
	Urea nitrogen - 63% (99%)*	Leukocyte esterase - 6% (5%)***
	Potassium - 62% (99%)*	Urine specific gravity - 6% (5%)***
CPT-4	Calcium - 53% (98%)***	Testosterone total - 29% (32%)***
	Anastomosis - 20% (98%)***	Surgery - 22% (14%)***
	Ultrasound, transrectal - 7% (65%)***	Ultrasound post-voiding residual urine/bladder capacity - 18% (29%)***
	Pelvic lymphadenectomy - 6% (100%)***	Urinalysis - 12% (44%)***
	Cystoplasty/cystourethroplasty - 6% (100%)***	Biopsy, prostate - 7% (31%)***

[1] Mean (standard deviation); [2] from ICD-9 on in-group and (total) percentages; [a] Multiple pairwise t-test; $^{*}p < 0.05$; $^{**}p < 0.01$; $^{***}p < 0.001$

PSA = Prostate-specific antigen

Supplementary Table 8: Most frequent terms for the two subgroups in the prostate cancer cohort.

	Malignant neoplasm of breast (female)	
	**Subgroup I** (N=5,971)	**Subgroup II** (N=1,993)
Age[1]	66.67 (14.27)^{*a}	62.86 (13.73)^{*a}
ICD-9[2]	Constipation (564.00) - 25% (93%)*	Lump or mass in breast (611.72) - 27% (29%)*
	Secondary malignant neoplasm (198.89) - 13% (93%)***	Abnormal mammogram (793.80) - 23% (37%)*
	Acquired absence of breast/nipple (V45.71) - 12% (92%)***	Carcinoma in situ of breast (233.0) - 15% (27%)^{ns}
	Antineoplastic chemotherapy (V58.11) - 7% (98%)***	Family history of malignant neoplasm of breast (V16.3) - 6% (28%)
	Mammogram for high-risk patient (V76.11) - 6% (63%)***	Abnormal findings on radiological examination of breast (793.89) - 4% (36%)***
Medication	Paracetamol - 50% (92%)***	Propofol - 27% (19%)***
	Ondansetron - 46% (87%)***	Fentanyl - 26% (16%)***
	Fentanyl - 45% (84%)***	Lidocaine - 25% (21%)***
	Oxycodone - 43% (91%)***	Midazolam - 22% (18%)***
	Propofol - 40% (81%)***	Ondansetron - 21% (13%)***
Lab test	Glucose - 67% (97%)***	Leukocytes - 7% (3%)***
	Leukocytes - 67% (97%)***	Glucose - 6% (3%)***
	Erythrocytes - 66% (97%)***	Platelets - 6% (3%)***
	Hemoglobin - 65% (97%)***	Erythrocytes - 6% (3%)***
	Hematocrit - 65% (97%)***	Mean corpuscular hemoglobin - 6% (3%)***
CPT-4	Surgery - 44% (81%)***	Mammography - 35% (32%)***
	Mastectomy, partial - 33% (78%)*	Ultrasound - 32% (27%)*
	Ultrasound - 30% (73%)*	Surgery - 30% (19%)***
	Unlisted chemotherapy - 23% (85%)***	Mastectomy, partial - 28% (22%)***
	Oncoprotein - 17% (85%)***	Mammography, bilateral - 26% (39%)***

[1] Mean (standard deviation); [2] from ICD-9 on in-group and (total) percentages; [a] Multiple pairwise t-test; $^{*}p < 0.05$; $^{**}p < 0.01$; $^{***}p < 0.001$

Supplementary Table 9: Most frequent terms for the two subgroups in the breast cancer cohort.

	Type 2 diabetes (Split 2)		
	**Subgroup I** (N=22,791)	**Subgroup II** (N=19,621)	**Subgroup III** (N=6,347)
Female/Male	12,121/10,652^{***a}	9,247/10,374^{***a}	2,651/3,692^{***a}
Age[1]	64.64 (14.50)$*^{b}$	67.17 (14.73)$*^{b}$	69.76 (11.35)$*^{b}$
ICD-9[2]	Hypertension (401.9) - 66% (46%)***	Edema (782.3) - 40% (48%)***	Coronary artery atherosclerosis (414.01) - 70% (29%)***
	Hyperlipidemia (272.4) - 53% (43%)***	Acute kidney failure (584.9) - 17% (82%)***	Coronary atherosclerosis (vessel) (414.00) - 67% (33%)***
	Chest pain (786.50) - 31% (46%)	Chronic kidney disease (585.9) - 16% (56%)***	Angina pectoris (413.9) - 49% (40%)***
	Obesity (278.00) - 22% (55%)***	Pain in limb (729.5) - 16% (42%)***	Abnormal result cardiovascular system function (794.39) - 39% (54%)***
	Hypercholesterolemia (272.0) - 19% (40%)***	Nephritis and nephropathy (583.81) - 14% (56%)***	Percutaneous transluminal coronary angioplasty (V45.82) - 30% (34%)***
Medication	Metformin - 54% (60%)***	Paracetamol - 58% (59%)***	Acetylsalicylic acid - 73% (27%)***
	Acetylsalicylic acid - 35% (50%)$^{***(\text{I vs II})}$	Glucagon - 45% (66%)***	Clopidrogel - 43% (34%)***
	Calcium - 27% (55%)***	Insulin lispro - 36% (61%)***	Intracoronary nitroglicerin - 41% (55%)***
	Paracetamol - 25% (30%)***	Vancomycin - 28% (84%)***	Bivalirudin - 39% (53%)***
	Cholesterol - 24% (69%)***	Furosemide - 27% (69%)***	Clopidrogel - 35% (33%)***
Lab test	Glucose - 52% (36%)***	Urea nitrogen - 82% (51%)***	Hematocrit - 81% (17%)$^{***(\text{III vs I})}$
	Creatinine - 48% (34%)***	Creatinine - 81% (50%)***	Mean corpuscular hemoglobin concentration - 80% (17%)$^{***(\text{III vs I})}$
	Leukocytes - 44% (32%)***	Bilirubin - 71% (56%)***	Chloride - 76% (15%)***
	Triglyceride - 37% (44%)***	ALT test - 68% (53%)***	Cholesterol - 50% (16%)***
	Cholesterol ratio - 37% (44%)*	Alkaline phosphatase - 66% (55%)***	Troponin I cardiac - 49% (23%)***
CPT-4	Calcium - 43% (37%)$^{***(\text{I vs II})}$	Potassium - 70% (57%)***	ECG; interpretation, report - 80% (18%)***
	Hemoglobin A1C - 41% (44%)***	Urea nitrogen - 68% (54%)***	Lipid panel - 50% (19%)***
	Glucose - 37% (38%)***	Creatinine - 68% (56%)***	Potassium - 44% (12%)***
	Lipid panel - 28% (38%)***	Urinalysis - 48% (69%)***	Troponin, quantitative - 41% (19%)*
	Lipoprotein, direct measurement - 24% (65%)***	Hepatic function panel - 25% (65%)***	C-reactive protein, high sensitivity - 34% (38%)***

[1] Mean (standard deviation); [2] from ICD-9 on in-group and (total) percentages; [a] Multiple pairwise chi-squared test; [b] Multiple pairwise t-test; $*\ p < 0.05$, $**\ p < 0.01$, $***\ p < 0.001$

ECG = Electrocardiogram; ALT = Alanine aminotransferase test

Supplementary Table 10: Most frequent terms for the three subgroups in the type 2 diabetes second split replication cohort.

	Parkinson's disease (Split 2)	
	**Subgroup I** (N=1,851)	**Subgroup II** (N=1,220)
Female/Male	799/1,051[a]	535/684[a]
Age[1]	71.39 (12.76)*[b]	75.65 (15.25)*[b]
ICD-9[2]	Anxiety state (300.00) - 24% (65%)**	Other malaise and fatigue (780.79) - 26% (53%)**
	Constipation (564.00) - 23% (57%)*	Chest pain (786.50) - 22% (68%)***
	Essential tremor (333.1) - 22% (79%)**	Coronary atherosclerosis (414.01) - 21% (79%)***
	Abnormality of gait (781.2) - 15% (48%)***	Atrial fibrillation (427.31) - 17% (85%)***
	Depressive disorder (311) - 14% (53%)***	Pleural effusion (511.9) - 17% (95%)***
Medication	Carbidopa/Levodopa combination - 49% (68%)***	Carbidopa - 47% (43%)*
	Amantadine - 17% (74%)***	Levodopa - 46% (42%)*
	Pramipexole - 15% (75%)***	Acetylsalicylic acid - 26% (72%)***
	Rasagiline - 14% (78%)***	Heparin - 23% (94%)***
	Selegiline - 12% (78%)***	Metoprolol - 18% (81%)***
Lab test	Glucose - 9% (15%)***	Erythrocytes - 77% (85%)***
	Leukocytes - 9% (15%)***	Mean corpuscolar hemoglobin - 75% (86%)***
	Creatinine - 9% (15%)***	Glucose - 75% (85%)***
	Erythrocytes - 9% (15%)***	Width - 75% (86%)***
	Urea nitrogen - 8% (15%)***	Leukocytes - 75% (85%)***
CPT-4	Unlisted psychiatric service or procedure - 29% (70%)***	Urea nitrogen - 60% (85%)***
	Surgery - 17% (48%)***	ECG; interpretation, report - 59% (82%)***
	MRI (brain, brain stem) - 16% (58%)	Urinalysis - 42% (87%)***
	CT head/brain - 5% (21%)***	Radiologic examination, chest - 38% (86%)***
	Implanted neurostimulator - 4% (68%)	Troponin, quantitative - 30% (85%)***

[1] Mean (standard deviation); [2] from ICD-9 on in-group and (total) percentages; [a] Multiple pairwise chi-squared test;

[b] Multiple pairwise t-test; $* \, p < 0.05$, $** \, p < 0.01$, $*** \, p < 0.001$;

CT = Computed tomography; ECG = Electrocardiogram; MRI = Magnetic resonance imaging

Supplementary Table 11: Most frequent terms for the two subgroups in the Parkinson's disease second split replication cohort.

	Alzheimer's disease (Split 2)		
	**Subgroup I** (N=1,422)	**Subgroup II** (N=1,461)	**Subgroup III** (N=267)
Female/Male	1,004/413^{a}	997/463^{a}	265/2^{***a}
Age[1]	71.64 (19.53)^{**b}	84.45 (10.61)^{**b}	42.12 (16.22)^{**b}
ICD-9[2]	Constipation (564.00) - 28% (45%)	Dementia w/o behavioral disturbance (294.10) - 39% (70%)***	Routine gynecological examination (V72.31) - 81% (53%)***
	Anxiety state (300.00) - 21% (56%)$^{***(\text{I vs II})}$	Altered mental status (780.97) - 26% (82%)***	Counseling (V65.40) - 57% (24%)***
	Memory loss (780.93) - 17% (62%)***	Persistent mental disorders (294.8) - 22% (80%)***	Osteoporosis (733.00) - 48% (20%)***
	Depressive disorder (311) - 13% (39%)***	Congestive heart failure (428.0) - 20% (75%)***	Family history of osteoporosis (V17.81) - 46% (25%)***
	Insomnia (780.52) - 10% (53%)$^{***(\text{I vs III})}$	Dementia with behavioral disturbance (294.11) - 19% (81%)***	Malignant neoplasm of uterus (179) - 35% (52%)***
Medication	Ergocalciferol - 28% (56%)***	Acetylsalicylic acid - 33% (70%)***	Ethinyl estradiol - 27% (65%)***
	Donepezil - 25% (45%)*	Donepezil - 30% (55%)*	Iron - 21% (12%)*
	Memantine - 17% (59%)***	Levofloxacin - 23% (83%)***	Gardasil - 15% (71%)***
	Vitamin B-12 - 14% (65%)***	Metoprolol - 23% (78%)***	Norethisterone - 12% (63%)***
	Docusate sodium - 12% (44%)$^{***(\text{I vs III})}$	Haloperidol - 20% (90%)***	Norgestimate - 6% (62%)*
Lab test	Glucose - 36% (30%)***	Erythrocytes - 83% (70%)***	Chlamydia/Gonorrhoeae amplified DNA - 16% (47%)***
	Erythrocytes - 36% (29%)***	Hematocrit - 82% (71%)***	HIV 1 - 4% (11%)
	Width - 35% (29%)***	Mean platelet volume - 82% (71%)***	Syphilis (rapid plasma reagin) - 4% (3%)$^{***(\text{III vs II})}$
	Platelets - 35% (30%)***	Urea nitrogen - 82% (72%)***	Hepatitis B surface antigen - 4% (10%)
	Hemoglobin - 35% (29%)***	Mean corpuscular hemoglobin concentration - 82% (71%)***	Hepatitis C virus ab - 4% (8%)
CPT-4	TSH - 38% (48%)**	Partial Thromboplastin Time Test - 59% (83%)***	Psychiatric service/procedure - 36% (18%)***
	ECG; interpretation, report - 34% (32%)***	Prothrombin time - 53% (85%)***	Calcium, ionized - 39% (14%)***
	Urea nitrogen - 29% (29%)***	X-ray chest - 49% (84%)***	Cytopathology, slides, cervical/vaginal - 31% (53%)***
	Creatinine - 26% (27%)***	Head/brain CT - 42% (77%)***	TSH - 21% (5%)***
	Psychiatric service/procedure - 20% (54%)***	Troponin, quantitative - 39% (80%)	Estradiol - 12% (40%)***

[1] Mean (standard deviation); [2] from ICD-9 on in-group and (total) percentages; [a] Multiple pairwise chi-squared test; [b] Multiple pairwise t-test; $*\ p < 0.05$, $**\ p < 0.01$, $***\ p < 0.001$;

ECG = Electrocardiogram; ab = antibodies; TSH = Thyroid-stimulating hormone; CT = Computed tomography

Supplementary Table 12: Most frequent terms for the three subgroups in the Alzheimer's disease second split replication cohort.

	Multiple myeloma (Split 2)			
	**Subgroup I** (N=191)	**Subgroup II** (N=891)	**Subgroup III** (N=502)	**Subgroup IV** (N=299)
Female/Male	108/83^{a}	473/418^{a}	230/272^{**a}	168/130^{**a}
Age[1]	61.97 (18.76)$^{**b(\text{I vs III/IV})}$	64.49 (12.44)$^{**b(\text{II vs III/IV})}$	67.24 (14.97)^{b}	67.30 (15.40)^{b}
ICD-9[2]	Other malaise and fatigue (780.79) - 19% (5%)***	Oth inflammatory/toxic neuropathy (357.89) - 48% (79%)**	Pleura effusion (511.9) - 42% (68%)***	Hyperlipidemia (272.4) - 35% (37%)**
	Disease of salivary glands (527.9) - 15% (7%)***	Unsp inflammatory/toxic neuropathy (357.89) - 38% (76%)***	Acute kidney failure (584.9) - 31% (68%)***	Nephritis and nephropathy (583.81) - 16% (21%)$^{***(\text{IV vs I/II})}$
	Constipation (564.00) - 15% (5%)**	Disorders of bone and cartilage (733.99) - 31% (59%)$^{***(\text{II vs I/IV})}$	Organ/tissue transplant (V42.9) - 31% (36%)$^{***(\text{III vs I/IV})}$	Dysuria (788.1) - 16% (18%)
	Fever (780.60) - 15% (4%)**	Disease of salivary glands (527.9) - 30% (67%)***	Renal failure (586) - 25% (43%)***	Monoclonal paraproteinemia (273.1) - 15% (17%)$^{**(\text{IV vs I/III})}$
	Counseling (V65.40) - 14% (5%)**	Organ/tissue transplant (V42.9) - 28% (59%)$^{***(\text{II vs I/IV})}$	Antineoplastic chemotherapy (V58.11) - 22% (37%)$^{***(\text{III vs I/IV})}$	Chronic kidney disease (585.9) - 13% (19%)*
Medication	Oxycodone - 12% (3%)**	Calcium - 59% (61%)***	Oxycodone - 63% (47%)**	Ergocalciferol - 35% (27%)*
	Lidocaine - 1% (4%)***	Dexamethasone - 45% (59%)***	Ondansetron - 56% (46%)**	Cholecalciferol - 31% (24%)***
	Acetylsalicylic acid 81 mg - 9% (3%)**	Bortezomib - 42% (70%)***	Diphenhydramine - 54% (52%)*	Atorvastatin - 15% (41%)***
	Dexamethasone - 9% (3%)***	Acetylsalicylic acid 81 mg - 38% (65%)**	Dexamethasone - 46% (34%)$^{***(\text{III vs I/IV})}$	Furosemide - 13% (14%)$^{***(\text{IV vs I/III})}$
	Paracetamol - 9% (2%)***	Lenalidomide - 37% (72%)***	Lorazepam - 39% (56%)***	Losartan - 13% (35%)***
Lab test	Leukocytes - 15% (2%)***	Width - 91% (57%)***	Mean corpuscular volume - 88% (31%)***	Glucose - 60% (12%)***
	Erythrocytes - 14% (2%)***	Mean platelet volume - 91% (57%)***	Chloride - 87% (31%)***	Leukocytes - 58% (12%)***
	Hematocrit - 14% (2%)*	Mean corpuscular hemoglobin - 91% (57%)***	Urea nitrogen - 87% (31%)***	Creatinine - 57% (12%)*
	Mean corpuscular volume - 14% (2%)***	Hemoglobin - 91% (56%)***	Leukocytes - 87% (30%)***	Protein - 57% (12%)***
	Platelets - 13% (2%)***	Protein - 90% (57%)***	Creatinine - 85% (30%)$^{***(\text{III vs I/IV})}$	Urea nitrogen- 57% (12%)***
CPT-4	Diagnostic/interventional CT - 23% (6%)**	Beta-2 microglobulin - 60% (73%)***	ECG; interpretation, report - 78% (46%)**	ECG; interpretation, report - 46% (16%)**
	PET limited area (Head/neck) - 19% (6%)***	Bone marrow, biopsy - 51% (65%)***	PTT - 76% (53%)***	Vitamin D - 30% (26%)***
	Surgery - 17% (7%)***	Nephelometry - 47% (70%)***	X-ray, chest - 66% (62%)***	Triglycerides - 29% (38%)***
	Psychiatric service/procedure - 15% (6%)*	Immunofixation - 46% (67%)***	Urinalysis - 65% (62%)***	Lipid panel - 25% (35%)*
	PET-CT (skull base to mid-thigh) - 12% (6%)**	Chemotherapy procedure - 41% (58%)$^{***(\text{II vs I/IV})}$	Phosphorus - 65% (54%)***	Cholesterol - 24% (54%)***

[1] Mean (standard deviation); [2] from ICD-9 on in-group and (total) percentages; ^{a} Multiple pairwise chi-squared test; ^{b} Multiple pairwise t-test; $^{*}p < 0.05$; $^{**}p < 0.01$; $^{***}p < 0.001$;

ECG = Electrocardiogram; CT = Computed tomography; PET = Positron emission tomography; PTT = Partial thromboplastin time

Supplementary Table 13: Most frequent terms for the four subgroups in the Multiple Myeloma second split replication cohort.

	Malignant neoplasm of prostate (Split 2)		
	**Subgroup I** (N=2,703)	**Subgroup II** (N=3,846)	**Subgroup III** (N=2,096)
Age[1]	68.71 (12.46)^{a}	70.92 (11.92)^{**a}	67.83 (14.49)^{a}
ICD-9[2]	Nocturia (788.43) - 28% (50%)***	Personal history of PC (V10.46) - 28% (77%)***	Palpitations (785.1) - 21% (51%)***
	Elevated PSA (790.93) - 20% (49%)***	Hyperlipidemia (272.4) - 25% (47%)***	Asthma (493.90) - 18% (51%)***
	Urinary frequency (788.41) - 17% (45%)$^{***(\text{I vs II})}$	Edema (782.3) - 23% (47%)***	Vitamin D deficiency (268.9) - 15% (72%)***
	Impotence of organic origin (607.84) - 16% (52%)***	Cardiac dysrhythmias (427.89) - 15% (69%)***	Cyanosis (782.5) - 14% (54%)***
	Urge incontinence (788.31) - 5% (52%)$^{***(\text{I vs II})}$	Pleural effusion (511.9) - 13% (87%)***	Neoplasm of colon (153.9) - 11% (52%)***
Medication	Midazolam - 15% (18%)***	Paracetamol - 68% (81%)***	Vitamin D3 - 17% (49%)***
	Tadalafil - 12% (47%)***	Oxycodone - 61% (82%)***	Fluticasone - 17% (61%)***
	Tamsulosin - 11% (23%)**	Ondansetron - 50% (82%)***	Atorvastatin - 17% (43%)***
	Testosterone - 8% (45%)$^{***(\text{I vs II})}$	Morphine - 50% (92%)***	Aerosol - 15% (53%)***
	Sildenafil - 10% (44%)$^{***(\text{I vs II})}$	Lidocaine - 47% (77%)***	Omeprazole - 10% (51%)***
Lab test	PSA total - 20% (33%)***	Glucose - 84% (68%)***	Glucose - 47% (21%)***
	PSA post-prostatectomy - 15% (37%)$^{***(\text{I vs III})}$	Leukocytes - 84% (72%)***	Cholesterol - 35% (49%)***
	Nitrite - 15% (18%)***	Urea nitrogen - 84% (72%)*	Hemoglobin A1C - 17% (52%)***
	PSA free - 11% (47%)***	Potassium - 84% (73%)$^{***(\text{I vs III})}$	Hepatitis C virus ab - 11% (53%)***
	Testosterone free - 6% (46%)$^{***(\text{I vs II})}$	Creatinine - 83% (72%)***	HIV 1 - 8% (55%)***
CPT-4	Surgery - 25% (25%)***	Calcium - 71% (72%)***	PSA total - 51% (23%)***
	Ultrasound post-voiding residual urine/bladder capacity - 28% (48%)***	ECG; interpretation, report - 43% (61%)$^{***(\text{II vs I})}$	PSA free - 52% (44%)***
	Ultrasound, transrectal - 16% (57%)***	Anastomosis - 33% (92%)$^{***(\text{II vs I})}$	ECG; interpretation, report - 41% (32%)$^{***(\text{III vs I})}$
	Urinalysis - 11% (60%)$^{***(\text{I vs II})}$	Urine culture, bacterial - 20% (69%)***	Surgery - 34% (26%)$^{***(\text{III vs I})}$
	MRI, pelvis - 9% (43%)**	Troponin, quantitative - 19% (90%)***	Spirometry - 14% (73%)***

[1] Mean (standard deviation); [2] from ICD-9 on in-group and (total) percentages; [a] Multiple pairwise t-test; $^{*}p < 0.05$; $^{**}p < 0.01$; $^{***}p < 0.001$

PSA = Prostate-specific antigen; ab = antibodies; ECG = Electrocardiogram; MRI = Magnetic resonance imaging

Supplementary Table 14: Most frequent terms for the three subgroups in the prostate cancer second split replication cohort.

	Malignant neoplasm of breast - female (Split 2)	
	**Subgroup I** (N=5,601)	**Subgroup II** (N=2,237)
Age[1]	66.98 (14.57)*[a]	61.94 (13.25)*[a]
ICD-9[2]	Personal history of malignant neoplasm of breast (V10.3) - 54% (79%)***	Lump or mass in breast (611.72) - 26% (33%)***
	Constipation (564.00) - 24% (92%)*	Abnormal mammogram (793.80) - 22% (43%)***
	Secondary malignant neoplasm (198.89) - 14% (91%)***	Other screening mammogram (V76.12) - 19% (44%)***
	Acquired absence of breast/nipple (V45.71) - 12% (89%)***	Carcinoma in situ of breast (233.0) - 15% (32%)*
	Antineoplastic chemotherapy (V58.11) - 7% (99%)***	Diffuse cystic mastopathy (610.1) - 10% (38%)***
Medication	Paracetamol - 50% (89%)***	Propofol - 28% (23%)***
	Fentanyl - 45% (80%)***	Fentanyl - 28% (20%)***
	Ondansetron 44% (83%)***	Midazolam - 24% (22%)***
	Oxycodone - 42% (88%)***	Lidocaine - 23% (23%)***
	Propofol - 38% (77%)***	Ondansetron - 23% (17%)***
Lab test	Leukocytes - 69% (97%)***	Leukocytes - 6% (3%)***
	Glucose - 69% (97%)***	Glucose - 6% (3%)***
	Hematocrit - 67% (97%)***	Width - 5% (3%)***
	Erythrocytes - 67% (97%)***	Mean corpuscular hemoglobin concentration - 5% (3%)***
	Width - 66% (97%)***	Erythrocytes - 5% (3%)***
CPT-4	Surgery - 43% (79%)***	Mammography - 33% (36%)***
	Mastectomy, partial - 34% (75%)***	Surgery - 30% (21%)***
	Ultrasound - 27% (68%)***	Mastectomy, partial - 28% (25%)***
	Unlisted chemotherapy - 24% (84%)***	Ultrasound, breast(s) - 24% (40%)***
	Oncoprotein - 16% (81%)***	Mammography, bilateral - 23% (42%)***

[1] Mean (standard deviation); [2] from ICD-9 on in-group and (total) percentages; [a] Multiple pairwise t-test; $^{*}p < 0.05$; $^{**}p < 0.01$; $^{***}p < 0.001$

Supplementary Table 15: Most frequent terms for the two subgroups in the breast cancer second split replication cohort.

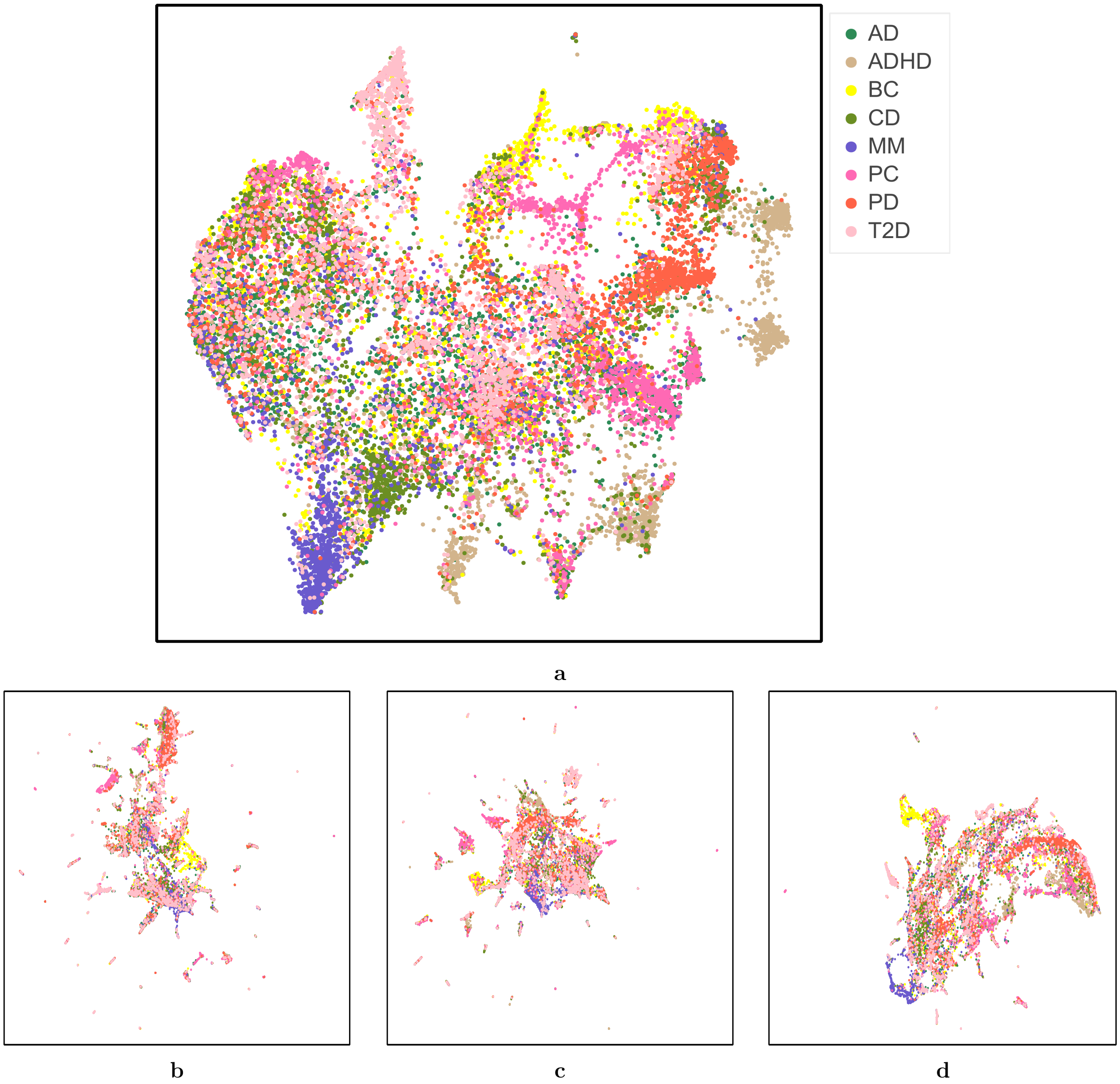

Supplementary Figure 1: Second split Uniform Manifold Approximation and Projection (UMAP) encoding visualization. ConvAE 1-layer CNN (**a**); SVD-RawCount (**b**); SVD-TFIDF (**c**); Deep Patient (**d**). AD = Alzheimer's disease; ADHD = Attention deficit hyperactivity disorder; BC = Breast cancer; CD = Crohn's disease; MM = Multiple myeloma; PC = Prostate cancer; PD = Parkinson's disease; T2D = Type 2 diabetes.

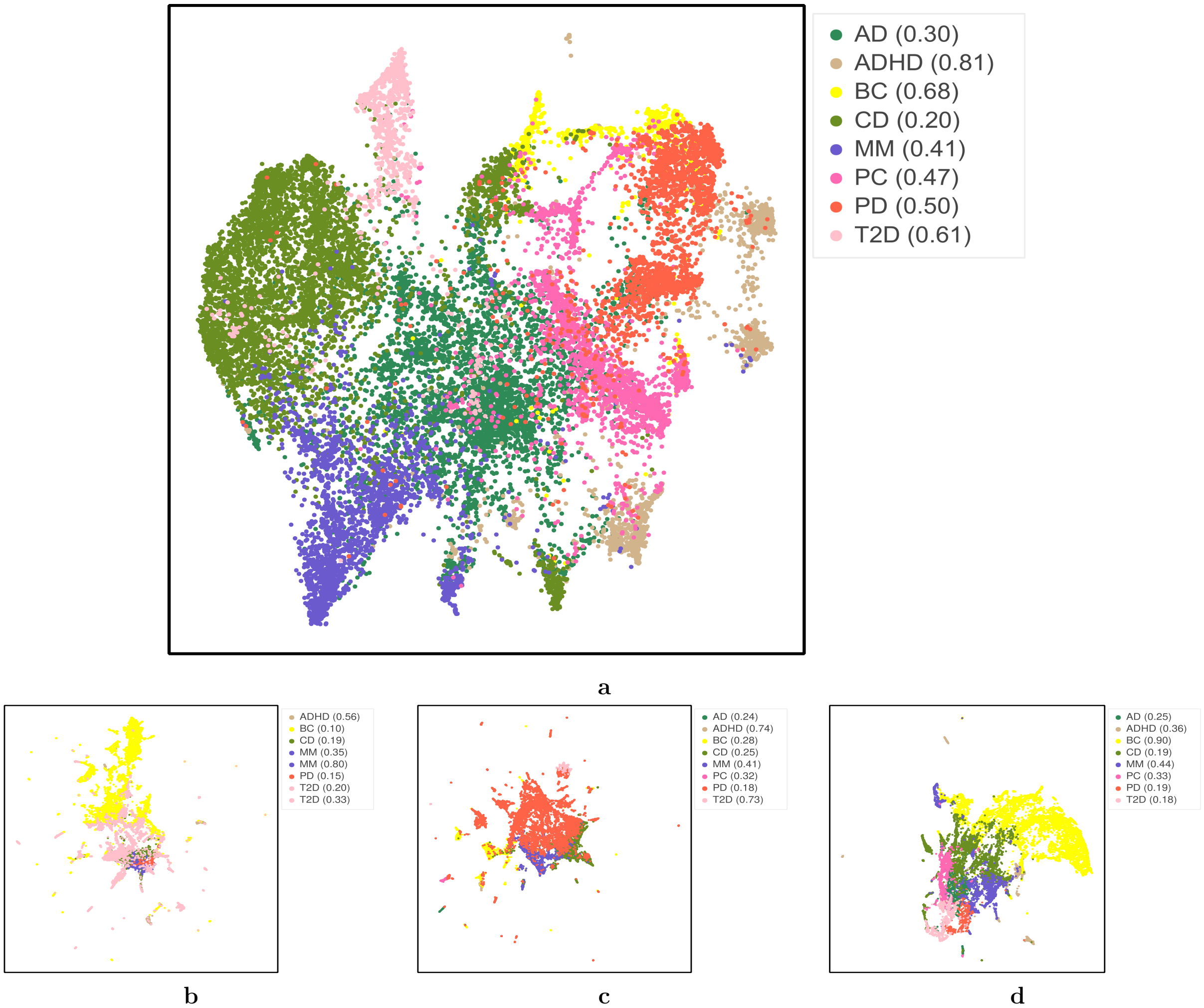

Supplementary Figure 2: Second split Uniform Manifold Approximation and Projection (UMAP) clustering visualization. ConvAE 1-layer CNN (**a**); SVD-RawCount (**b**); SVD-TFIDF (**c**); Deep Patient (**d**). AD = Alzheimer's disease; ADHD = Attention deficit hyperactivity disorder; BC = Breast cancer; CD = Crohn's disease; MM = Multiple myeloma; PC = Prostate cancer; PD = Parkinson's disease; T2D = Type 2 diabetes.

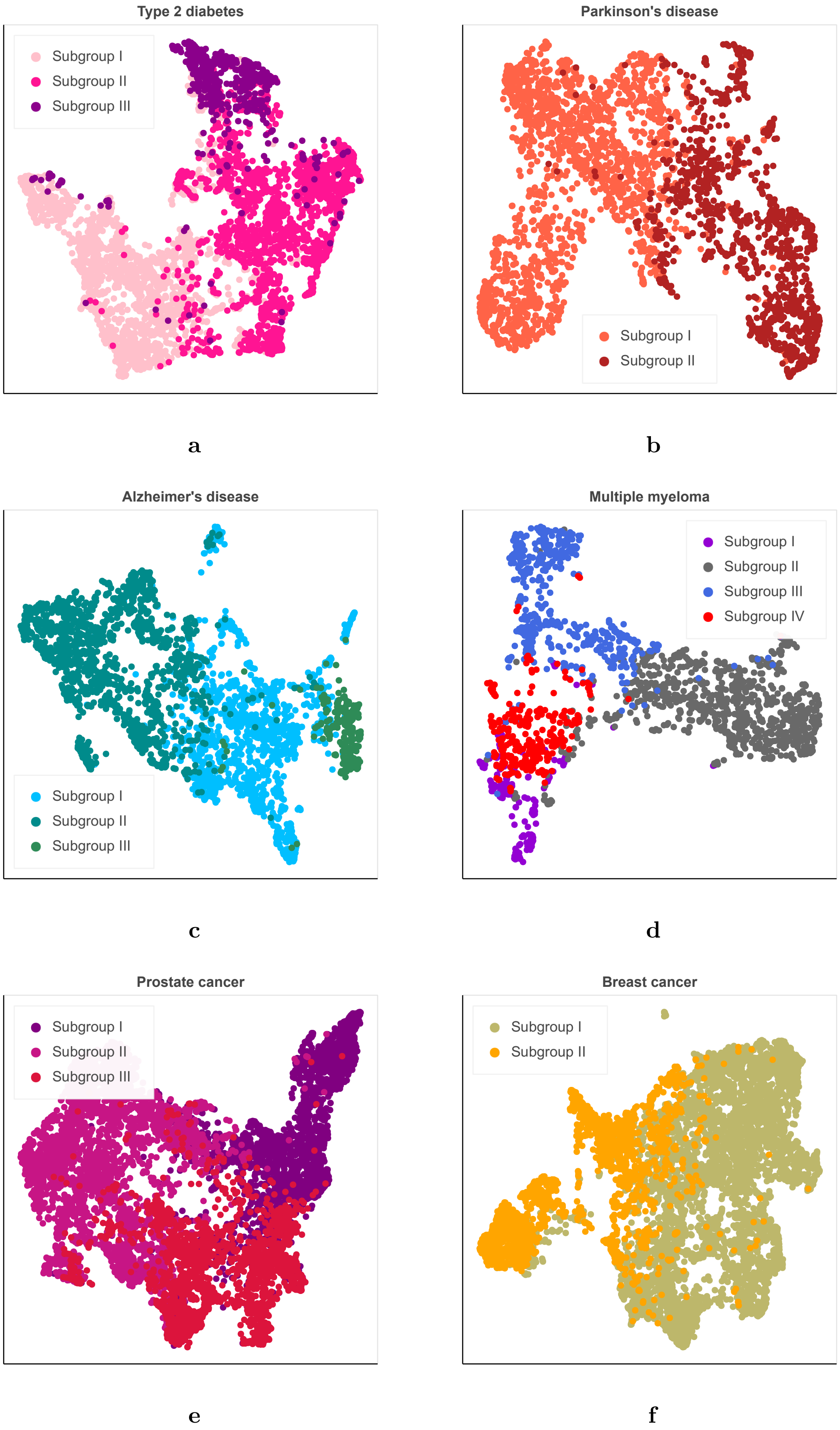

Supplementary Figure 3: Complex disorder subgroups identified in the replication set. A subsample of 5,000 patients with T2D is displayed in Figure (**a**).
